# Comparative evaluation of p5+14 with SAP and peptide p5 by dual-energy SPECT imaging of mice with AA amyloidosis

**DOI:** 10.1038/srep22695

**Published:** 2016-03-03

**Authors:** Emily B. Martin, Angela Williams, Tina Richey, Alan Stuckey, R. Eric Heidel, Stephen J. Kennel, Jonathan S. Wall

**Affiliations:** 1 Department of Medicine, University of Tennessee Graduate School of Medicine, 1924 Alcoa Highway, Knoxville, TN, 37920, USA; 2 Department of Radiology, and University of Tennessee Graduate School of Medicine, 1924 Alcoa Highway, Knoxville, TN, 37920, USA; 3 Department of Surgery, University of Tennessee Graduate School of Medicine, 1924 Alcoa Highway, Knoxville, TN, 37920, USA.

## Abstract

Amyloidosis is a protein-misfolding disorder characterized by the extracellular deposition of amyloid, a complex matrix composed of protein fibrils, hyper-sulphated glycosaminoglycans and serum amyloid P component (SAP). Accumulation of amyloid in visceral organs results in the destruction of tissue architecture leading to organ dysfunction and failure. Early differential diagnosis and disease monitoring are critical for improving patient outcomes; thus, whole body amyloid imaging would be beneficial in this regard. Non-invasive molecular imaging of systemic amyloid is performed in Europe by using iodine-123-labelled SAP; however, this tracer is not available in the US. Therefore, we evaluated synthetic, poly-basic peptides, designated p5 and p5+14, as alternative radiotracers for detecting systemic amyloidosis. Herein, we perform a comparative effectiveness evaluation of radiolabelled peptide p5+14 with p5 and SAP, in amyloid-laden mice, using dual-energy SPECT imaging and tissue biodistribution measurements. All three radiotracers selectively bound amyloid *in vivo*; however, p5+14 was significantly more effective as compared to p5 in certain organs. Moreover, SAP bound principally to hepatosplenic amyloid, whereas p5+14 was broadly distributed in numerous amyloid-laden anatomic sites, including the spleen, liver, pancreas, intestines and heart. These data support clinical validation of p5+14 as an amyloid radiotracer for patients in the US.

Amyloid deposits are composed of protein fibrils, in association with hyper-sulphated glycosaminoglycans and serum proteins such as serum amyloid P component (SAP)^1^. The relentless deposition of extracellular amyloid leads to the disruption of tissue architecture^2^,^3^, cytotoxicity^4^ and, eventually, organ dysfunction and failure. Systemic amyloidosis is an orphan disease, with ~3500 new diagnoses annually in the US. In these patients, amyloid can involve any visceral tissue, but cardiac and renal involvement is associated with the highest rates of mortality^1^. Due to their rarity and the heterogeneous clinical presentation, early and accurate diagnosis of systemic amyloid diseases remains challenging^5^. Expert opinions from physicians and researchers alike suggest that early diagnosis is critical to improving patient outcomes^6^,^7^. Diagnosis of amyloidosis currently relies on examination of biopsy-derived tissue samples stained with Congo red. The presence of amyloid yields green birefringence when observed microscopically with cross-polarized illumination^8^,^9^. This technique is dated, yet remains the diagnostic standard. However, the staining procedure and interpretation are not trivial, and biopsies are prone to sampling error, all of which can lead to false negative findings. Additionally, a positive diagnosis based on tissue Congophilia does not inform the extent of whole body amyloid burden in patients, and acquisition of multiple organ biopsies is not practical and may contribute to morbidity.

Obtaining a complete representation of the amyloid load in patients can confirm diagnosis, inform prognosis and assist in disease monitoring. At present, the gold standard for systemic amyloid imaging, which is routinely used only in Europe, involves gamma scintigraphy using iodine-123-labelled SAP as the radiotracer^10^,^11^,^12^. Although effective, this technique is not approved by the FDA for use in the US; therefore, an alternative imaging approach is necessary. Furthermore, it has not shown to be effective for clinical detection of amyloid in the heart^13^,^14^, an organ that is involved in more than 50% of patients with light chain-associated (AL) amyloidosis – the most common form of visceral amyloid disease^7^. To this end, we have developed synthetic, amyloid-reactive, poly-basic peptides which preferentially bind amyloid via electrostatic interactions^4^. These peptides have been used to specifically target and stain human AL and transthyretin-associated (ATTR) amyloid deposits in formalin-fixed tissue sections *in vitro*^15^,^16^. Additionally, peptide reactivity with inflammation-associated (AA) amyloid in a transgenic mouse model has been documented by using single photon emission computed tomography (SPECT) imaging and microautoradiography^4^,^16^,^17^,^18^. Notably, two α-helical peptides with a Lys-Ala-Gln-Lys-Ala-Gln-Ala – heptad repeat, designated p5 and p5+14, have been well characterised^4^,^15^,^16^,^17^,^18^,^19^,^20^ (Fig. 1). Of these, peptide p5+14 has a higher affinity for amyloid, due to the increased electropositivity, and has shown exceptional promise as an amyloid-imaging agent^18^. Plans to evaluate this reagent in a Phase I imaging clinical trial of patients with AL amyloidosis are underway.

Herein, we report a series of comparative effectiveness studies that contrast peptide p5+14 with p5 and the clinical standard, SAP, in individual mice with AA amyloidosis by using dual-energy SPECT imaging and quantitative, spillover-corrected tissue biodistribution measurements^20^. We have shown, *in vitro*, that peptide p5+14 bound more avidly to human AL amyloid fibrils, as compared to p5, and it also exhibited significantly greater uptake in certain visceral organs of AA mice. Furthermore, when compared to SAP, which was seen mostly in the liver and spleen, p5+14 localised in numerous AA amyloid-laden anatomic sites, notably the pancreas and heart of diseased mice.

## Results

**Figure 1:**
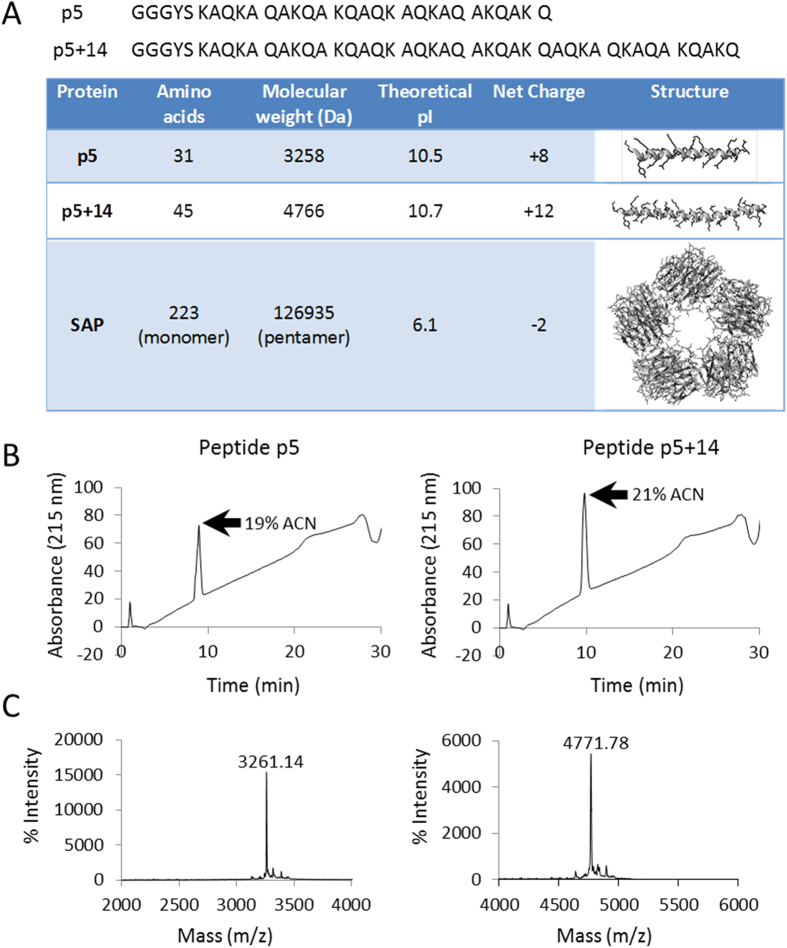
Physical characteristics of amyloid-reactive peptides p5 and p5+14, and protein SAP. (**A**) The primary structure of peptides p5 and p5+14 show the characteristic heptad repeat. Physical properties for all 3 proteins were calculated using the Protparam software (http://web.expasy.org/protparam/). Secondary structures of peptides were predicted using the online prediction program, I-TASSER^40^. The x-ray crystal structure of SAP has been determined, PDB# 1SAC. Chromatograms from HPLC purification (**B**) and mass spectrometry spectra (**C**) of peptides p5 and p5+14. Masses for peptides p5 and p5+14 represent [M + 3H]^+3^ and [M + 5H]^+5^, respectively.

**Figure 2:**
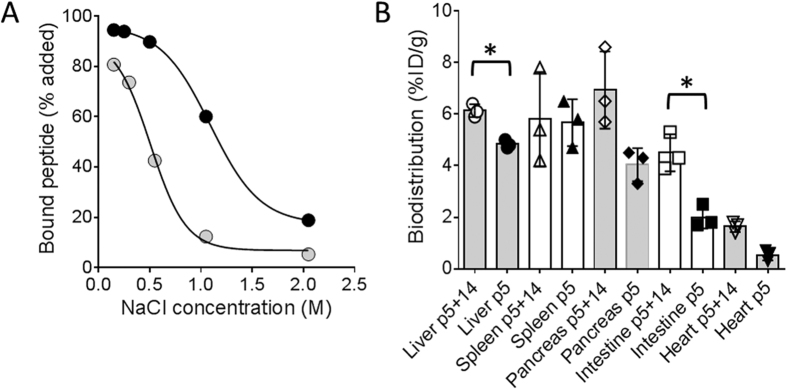
Comparison of peptide p5 and p5+14 amyloid reactivity *in vitro* and *in vivo*. (**A**) Binding of ^125^I-p5 (grey circles) and ^125^I-p5+14 (black circles) to rVλ6Wil fibrils, by peptide binding assay, in solutions of increasing ionic strength (mean ± SD; SD was <10% for all points, and error bars are not visible). The data were fit with a sigmoid equation using Prism. (**B**) Dual-energy tissue biodistribution of ^125^I-p5+14 and ^99m^Tc-p5 in AA mice. Bars represent the mean %ID/g (*n* = 3) with SD. Individual data points for each mouse are shown. Data were analysed by using an independent t-test with a Bonferroni corrected α value of 0.01, where *p < 0.01.

Both peptides were purified as a single species by reverse phase HPLC and had appropriate masses, with additional protons [M + 3H]^+^^3^ and [M + 5H]^+^^5^, as determined by matrix-assisted laser desorption/ionisation time-of-flight (MALDI-TOF) mass spectrometry (Fig. 1). The peptides and SAP were readily radiolabelled with >90% radiopurity following chromatographic purification, as evidenced by SDS-PAGE and phosphorimaging. Initially, we compared the reactivity of peptides p5 and p5+14 for their ability to bind amyloid *in vitro* and *in vivo*. We hypothesised that the increased net charge of p5+14 (Fig. 1) would confer enhanced amyloid reactivity due to increased electrostatic interactions. Binding assays with ^125^I-labelled peptides added to synthetic rVλ6Wil and IAPP fibrils, as well as human ALκ4 amyloid extract and murine AA liver homogenate, revealed, in every case, a statistically significant increase in binding of ^125^I-p5+14 as compared to ^125^I-p5 (*p* < 0.05 using an unpaired *t*-test; Table 1). Binding of p5+14 to murine AA-laden liver homogenate (*p* = 0.002) and rVλ6Wil (*p* = 0.02) was ~15% greater; however, ALκ4 binding was increased by 40% (*p* = 0.0006), and there was a two-fold increase in reactivity with IAPP fibrils (*p* = 0.001). To assess the relative electrostatic avidity of each peptide for rVλ6Wil fibrils, binding assays were performed in milieu of increasing ionic strength (Fig. 2A). The NaCl concentration required to decrease binding by 50% (IC_50_) was 0.5 M for ^125^I-p5 but increased to ~1.2 M for peptide ^125^I-p5+14, indicating that the extra 4 lysine residues had conferred enhanced electrostatic avidity for the fibrils (Fig. 2A).

Dual-energy tissue biodistribution measurements were performed to compare the reactivity of ^125^I-p5 and ^99m^Tc-p5+14 in individual AA-laden mice (Fig. 2B). Retention of ^99m^Tc-p5+14 was significantly greater (*p* < 0.01) in amyloid-laden liver and intestines as compared to peptide ^125^I-p5, and it was comparable in all other organs evaluated (Fig. 2B).

**Figure 3:**
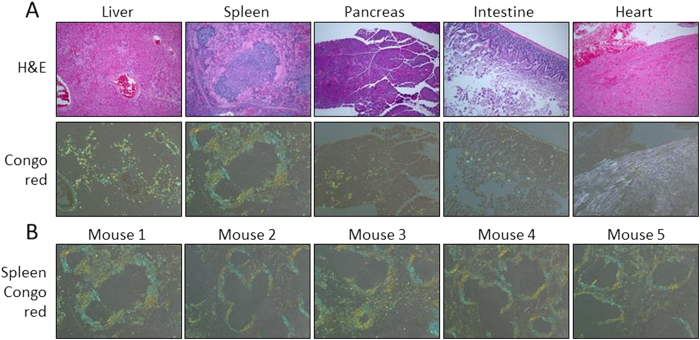
Systemic distribution of AA amyloid in H2/IL-6 mice. (**A**) Tissue sections, stained with H&E or Congo red, from a representative mouse indicate the presence of severe systemic amyloid in AA mice, as evidenced by the blue-gold birefringence, indicative of amyloid, seen in the Congo red-stained sections. (**B**) Each of the five AA mice had severe AA amyloid as evidenced by the presence of Congo red-birefringent deposits in a representative tissue (spleen). Original objective magnification 10×.

We next compared peptide p5+14 with the gold-standard clinical imaging agent, SAP, by using dual-energy SPECT imaging. Transgenic H2/IL-6 mice were induced to develop severe systemic AA amyloidosis by injection of a suspension of extracted murine splenic AA amyloid. The presence of amyloid in the mice was confirmed by examination of tissue sections using the amyloidophilic dye, Congo red (Fig. 3). Each of the five AA amyloid mice was shown to have amyloid disease.

**Figure 4:**
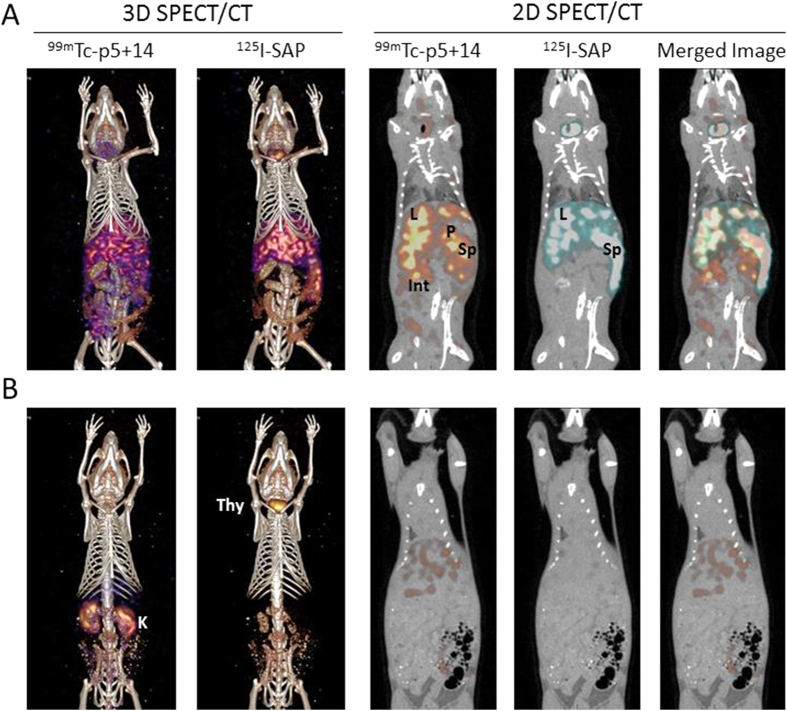
Dual-energy SPECT/CT images of ^125^I-SAP and ^99m^Tc-p5+14 in representative AA amyloid-laden and WT mice. Three-dimensional and 2-D coronal images of the distribution of ^99m^Tc-p5+14 and ^125^I-SAP in individual mice with AA amyloidosis (**A**) and a healthy WT control (**B**). Radioactivity is false coloured red – blue (3D images) and red or blue for ^99m^Tc-p5+14 and ^125^I-SAP, respectively in the 2D images. Where: L, liver; P, pancreas; Sp, spleen; Int, intestines; K, kidney; Thy, thyroid. Two mice, chosen randomly, from each set of five AA and five WT mice were imaged.

SPECT/CT imaging of ^125^I-SAP and ^9m^Tc-p5+14 in AA mice revealed organ-specific differences in amyloid uptake of the two radiotracers (representative subject shown in Fig. 4A). In 3D representations of the image data, ^99m^Tc-p5+14 was observed relatively uniformly distributed throughout the visceral organs. This contrasted with the binding of ^125^I-SAP, which manifest principally as hepatosplenic uptake (Fig. 4A). Two-dimensional representations of the SPECT/CT image confirmed visual uptake of ^99m^Tc-p5+14 in the liver (L), spleen (Sp), pancreas (P), and intestines (Int) and almost exclusive uptake of ^125^I-SAP by amyloid in the liver and spleen (Fig. 4A). In these experiments, there was no visual evidence of uptake of either radiotracer in heart, which is known to have only scant, diffuse deposits of amyloid in this model.

In amyloid-free, WT animals (Fig. 4B), radioactivity was observed in the kidneys (K) of mice injected with ^99m^Tc-p5+14 due to the accumulation of the ^99m^Tc in the renal cortex associated with catabolism of the peptide. In contrast, radioiodide was observed in the thyroid (Thy) of mice that received ^125^I-SAP (Fig. 4B). This was due to liberation of radioiodide during dehalogenation and catabolism of the SAP in the liver and organification of the liberated iodide by the thyroid. Notably, there was no specific uptake of either radiotracer in amyloid-free organs.

**Figure 5:**
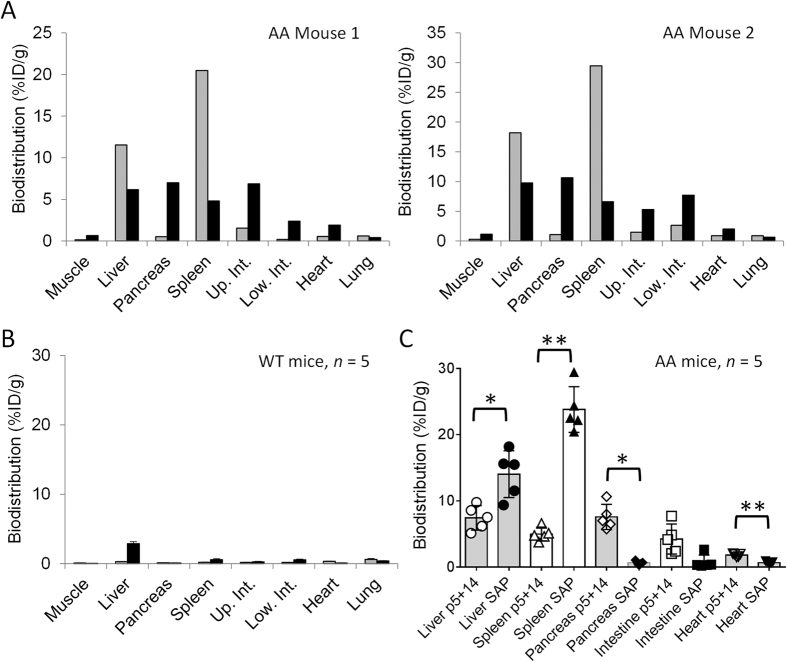
Dual-energy biodistribution data for ^125^I-SAP and ^99m^Tc-p5+14 in two AA amyloid-laden mice and a representative WT animal. Radiotracer biodistribution at 24 h (SAP; grey bars) and 4 h (p5+14; black bars) post injection, in two representative female mice with AA amyloidosis at 5 wk post-AEF (**A**), and the mean ± SD values of five WT animals (**B**). Data are expressed as cross-over corrected %ID/g. Up. Int. and Low. Int. represents the upper and lower intestines, respectively. (**C**) Statistical analysis of tissue biodistribution data. Bars represent the mean (*n* = 5) with SD. Individual data points for each mouse are shown (open symbols = p5+14; filled symbols = SAP). Data for liver, spleen, intestine and heart were analysed by using an independent t-test and the pancreas with a Mann Whitney U test. Significance was established using Bonferroni corrected α value of 0.01, where *p < 0.01, **p < 0.001.

Quantitative comparison of ^125^I-SAP and ^99m^Tc-p5+14 uptake in amyloid-laden organs was achieved by using dual-energy, spillover-corrected tissue biodistribution measurements (Fig. 5 and Table 2). As predicted by the SPECT imaging data, ^99m^Tc-p5+14 and ^125^I-SAP accumulated in organs containing amyloid deposits–liver, pancreas, spleen, and intestines (Fig. 5A; representative individual mice). However, no retention was observed in amyloid-free organs in WT mice (Fig. 5B).

The uptake of ^125^I-SAP was ~4-fold greater in the spleen as compared to ^99m^Tc-p5+14 in each mouse studied (*p* < 0.001). In contrast, ^99m^Tc-p5+14 was retained in significantly greater amounts by amyloid in the pancreas (*p* = 0.009) and heart (*p* < 0.001), as compared to ^125^I-SAP. Notably, uptake of ^125^I-SAP by pancreatic AA amyloid was remarkably low (<1%ID/g), whereas, the mean uptake of ^99m^Tc-p5+14 in this organ was 7.6% ID/g (*n* = 5 mice). The binding of ^99m^Tc-p5+14 in the heart was significantly greater than that of ^125^I-SAP (*p* < 0.001); however, cardiac AA amyloid is difficult to detect in SPECT images due to the diffuse and scant nature of the deposits in this organ. With the exception of the liver, no direct correlation between the uptake of SAP and p5+14 was demonstrated by Pearson correlation analysis in the organs evaluated, which suggests that the two radiotracers may bind independent amyloid-associated ligands (Table 2).

## Discussion

There are currently no methods in the US for imaging the whole-body amyloid burden in patients with systemic amyloidosis. Although certain bone-seeking agents and Aβ amyloid-imaging tracers have shown promise in detecting cardiac amyloid disease, their use is limited, and there is no evidence that they are capable of detecting amyloid in all anatomic sites^21^,^22^,^23^,^24^. Given the heterogeneity of organ involvement in patients with systemic amyloidosis, there remains a need to develop agents capable of imaging amyloid in multiple anatomic sites—preferably with pan-amyloid reactivity. In Europe, systemic amyloid is effectively imaged by planar gamma scintigraphy and SPECT using human plasma-derived SAP radiolabelled with ^123^I^10^,^11^,^12^. This reagent is not approved for use by the US Food and Drug Administration, possibly due to their requirement for stringent, anti-viral procedures for human-derived, biological molecules, which inactivate the amyloidophilic activity of SAP. Therefore, we have developed a series of synthetic, poly-basic, heparin-binding peptides with high affinity and specificity for the hyper-sulphated glycosaminoglycans found ubiquitously in amyloid deposits^4^,^16^,^25^,^26^,^27^. Additionally, these peptides also interact directly with amyloid fibrils regardless of the constituent protein from which they are formed^15^,^18^. The goal of this study was to directly compare the amyloid binding activity of peptides p5 and p5+14, as well as p5+14 with the current clinical standard, SAP.

The peptide p5 was identified in a screen of seven naturally-occurring and synthetic heparin-reactive peptides as a specific amyloid reactive peptide as evidenced by SPECT/CT of AA amyloid mice, biodistribution studies and microautoradiography^16^. The interaction with amyloid is driven principally by electrostatic interactions; however, p5 does not bind the abundant heparan sulphate proteoglycans expressed on the cell surface and extra-cellular matrices of healthy tissues^16^. Based on these initial observations, we hypothesised that peptides with enhanced propensity for α-helicity and with increased positive charge might improve the observed peptide-amyloid interactions, which are deemed to be dependent on peptide secondary structure and electrostatic interactions^28^.

With this in mind, peptide p5+14 was constructed as an extended variant of p5 with an additional four lysine residues. This peptide was developed as an enhanced amyloid-targeting agent for use as a molecular imaging tracer in patients with systemic amyloidosis^18^. Peptide p5+14 has been shown to bind numerous synthetic amyloid fibrils (AL, Aβ, and AIAPP) as well as human ALκ and ALλ amyloid extracts *in vitro*^18^. In addition, when radiolabelled, p5+14 preferentially bound AA amyloid in a murine mouse model and did not accumulate in healthy, amyloid-free tissues^18^. Translation of the p5+14 peptide as a clinical imaging agent for patients with AL and transthyretin-associated systemic amyloidosis is currently being supported by the *Science Moving towArds Research Translation and Therapy* (SMARTT) Program at the National Heart, Lung and Blood Institute of the NIH.

The data presented herein indicate that peptide p5+14 has significantly enhanced reactivity, compared to p5, with AA amyloid *in vivo* as well as with AL and AIAPP amyloid fibrils and extracts *in vitro*. The increased electropositive charge resulted in more avid binding to synthetic fibrils, as evidenced by the stability of the peptide-fibril interaction in milieu of increasing ionic strength (Fig. 2A). The enhanced amyloid-reactivity due to increasing charge likely contributes to the increased uptake and retention of this peptide by amyloid *in vivo*. However, net positive charge alone is insufficient to explain the specific reactivity of these peptides with amyloid because similarly highly charged peptides, such as protamine, do not exhibit equivalent specificity *in vivo*^16^. Rullo *et al*. demonstrated that α-helical peptides with lysine residues aligned in an array on one face of the helix bound heparin more effectively than equally charged peptides with non-linear spacing of lysine residues^28^. We hypothesised, therefore, that the increased lysine content with a stringent heptad repeat, in the context of an α-helical secondary structure, would render p5+14 a more effective amyloid binding agent as compared to peptide p5.

Radioiodinated SAP is the “gold standard” radiotracer used, in Europe, for gamma scintigraphic imaging of amyloid in patients. To validate the effectiveness of our ^99m^Tc-p5+14 imaging agent compared to ^125^I-SAP, we examined their amyloid reactivity using dual-energy SPECT imaging, which is a powerful technique for comparative evaluation of two radiotracers in individual animals. This is achieved by resolving the low- and high-energy radionuclides in the SPECT data. Dual-energy SPECT imaging circumvents the variability of amyloid deposition in individual mice since both ^125^I-SAP and ^99m^Tc-p5+14 were injected into and evaluated in the same subject. The uptake of ^125^I-SAP in the mice was principally hepatosplenic with significantly higher retention in the spleen and liver, relative to ^99m^Tc-p5+14. The molecular binding site of SAP on amyloid remains enigmatic, although it has been shown *in vitro* to bind both heparin^29^ and synthetic fibrils^30^, similar to the binding profile of peptide p5+14. It is unlikely that SAP could compete with p5+14 for amyloid binding sites in our imaging experiments, since only microgram quantities of each radiotracer was injected, and the amyloid load is extensive (we estimate ~10–20% of the mass of the spleen based on histological staining of tissue sections). In this mouse model, the organ uptake profiles suggest that SAP and p5+14 bind different sites in amyloid deposits (Fig. 4). One explanation for this observation is that, in the spleen, the SAP-reactive ligand is abundant and extensively available for binding—the same is not true for the p5+14 amyloid binding site. Alternatively, pharmacokinetic or other physical barriers, not encountered by SAP, may hinder accessibility and uptake of p5+14 in the splenic amyloid deposits. Although the precise mechanism underlying this disparity remains enigmatic, the existence of discrete binding sites for SAP and p5+14 is also supported by the finding that pancreatic AA amyloid, in these mice, preferentially bound p5+14, but little or no SAP was observed in this organ. This finding is consistent with previous imaging studies with SAP in this mouse model^20^,^31^. It is interesting to speculate that, even within an individual subject with systemic amyloidosis, there are tissue-specific amyloid “phenotypes” which may be related to the sulphation pattern of the glycosaminoglycans co-deposited in the amyloid or differences in the morphology of the fibrils^32^. Regardless, the image and biodistribution data indicate that ^99m^Tc-p5+14 more accurately defines the organ distribution of amyloid deposits in the AA mice as compared to ^125^I-SAP.

We have previously demonstrated specific uptake of synthetic peptides, p5 and p5+14, in all amyloid deposits of AA mice by using microautoradiography^16^,^18^. In the present study, ^99m^Tc-p5+14 was compared directly with p5 and exhibited superior affinity for amyloid. In comparison to SAP, radiolabelled peptide p5+14 provided more enhanced images of amyloid deposits in numerous anatomic sites (Fig. 4). Thus, peptide p5+14 has the potential to provide effective whole body imaging of systemic amyloid load that is comparable to, and perhaps in certain tissues superior to, that afforded by SAP.

## Materials and Methods

### Animals

Transgenic H2-L^d^-huIL-6 Balb/c mice (H2/IL-6) constitutively express the human IL-6 transgene leading to chronic, severe inflammation and the overproduction of acute phase reactants, such as serum amyloid protein A, the precursor protein of systemic, inflammation-associated (AA) amyloidosis^31^,^33^. Amyloid deposition was induced in 8–wk old female mice by IV injection of 100 μg of amyloid enhancing factor (AEF – an insoluble preparation of amyloid fibrils isolated from the spleen of mice with AA amyloidosis) suspended in sterile phosphate buffered saline (PBS)^34^. All animal procedures were performed in accordance with protocols approved by the University of Tennessee Animal Care and Use Committee. The University of Tennessee is an Association for Assessment and Accreditation of Laboratory Animal Care International (AAALAC-I) accredited institution.

### Radiotracer preparation

Human SAP was isolated from autopsy-derived amyloid laden tissues, as previously described^20^. Briefly, human amyloid-laden spleen was suspended at ~100 mg/mL using a Polytron homogenizer (Brinkmann Instruments, Westbury, New York) at setting 6, for 10 sec in tris-buffered saline (TBS)/2 mM CaCl_2_. The insoluble material was then pelleted by centrifugation at 10,000 × g for 30 min and washed again, by centrifugation. The sample was then homogenised again in the presence of TBS/10 mM ethylenediaminetetraacetic acid (EDTA). After a final centrifugation as above, CaCl_2_ was added to the supernatant, containing crude human SAP to achieve a final concentration of 20 mM and incubated for 2 hours with 5 mL *O*-phosphoryl-ethanolamine (PE)-conjugated-Sepharose beads (prepared by mixing 113 mg PE with 50 mL of activated ECH-Sepharose [GE Healthcare] at pH 4.5 overnight) at room temperature. The beads were then washed 2 times with 100 mL of TBS/2 mM CaCl_2_ and the human SAP eluted by addition of TBS/10 mM EDTA. Fractions containing SAP were pooled and dialysed against 10 volumes of PBS. The product was shown to be >95% pure by examination of Coomassie-stained SDS-PAGE gel profiles.

Purified SAP (~100 μg) was radioiodinated by addition of 2 mCi of ^125^I (PerkinElmer, Waltham, MA) in the presence 10 μg chloramine T, for 2 min^35^. The reaction was quenched by adding 20 μg of sodium metabisulphite. Unbound radioisotope was separated from the product by gel filtration chromatography on Ultragel Aca-34 resin, using PBS/0.1% gelatin as the mobile phase. Fractions of ~0.5 mL were collected and the radioactivity in each measured by gamma counting—the three fractions with the highest counts were pooled.

Peptides p5 and p5+14 were synthesised commercially (Keck Laboratories, New Haven, CT) and purified, in house, by reverse-phase HPLC using an acetonitrile (ACN), 0.05% trifluoroacetic acid mobile phase with a flow rate of 1 mL/sec (2%/min increase in ACN). Samples of peptide p5 and p5+14 isolated from the HPLC analysis were dried and resuspended in analyte solvent (30% ACN, 0.01% TFA in water) at ~0.2 mg/mL in preparation for (+)MALDI-TOF analysis. α-Cyano-4-hydroxycinnamic acid (CHCA) was suspended in matrix solvent (50% ACN, 0.3% TFA in water) at a final concentration of 8 mg/mL. Four μL of analyte suspension (peptide) and 24 μL of matrix solution were mixed, and 1 μL of the mixture was spotted into the MALDI plate. Mass spectrometry was carried out on a Voyager DE-PRO mass spectrometer (Applied Biosystems, Boston, MA, USA). Typically, mass spectrometric spectra were obtained in linear mode with a laser power of 2390 kW.cm^−2^. All MALDI spectra were externally calibrated with known peptide standards.

The peptides were radiolabelled with ^125^I essentially as described above, but purified by gel filtration using a Sephadex G-25 size exclusion matrix (PD10; GE Healthcare)^16^,^20^. Conjugation of peptides with ^99m^Tc was achieved as follows. To 20 μL of 0.15 N NaOH was added, 20 μL (~40 μg) of peptide solubilised in water and 10 μL of 100 μg/mL SnCl_2_ freshly prepared in 0.01 N HCl followed by 2 mCi of ^99m^Tc0_4_^−^ in ~100 μL saline (Cardinal Health, Knoxville, TN). After 15 min reaction time at RT, the product was purified using a PD10 column as described above^36^. The radiochemical purity of all products was determined qualitatively by SDS polyacrylamide gel electrophoresis analysed by phosphor imaging (Cyclone Storage Phosphor System, PerkinElmer, Shelton, CT).

### SPECT/CT Imaging

Dual-energy comparative uptake experiments were performed in individual AA mice (5 wk post amyloid induction with AEF) and wild type mice (*n* = 5 per group). All SPECT/CT imaging was performed using an Inveon trimodality SPECT/PET/CT platform^37^ (Siemens preclinical, Knoxville, TN). Image data were acquired and reconstructed using Inveon Acquisition Workplace (IAW) ver. 2.0.

For the comparison of ^125^I-SAP and ^99m^Tc-p5+14, mice were administered ^125^I-SAP (45 μCi, 8 μg) and 20 h thereafter received ^99m^Tc-p5+14 (110 μCi, 5 μg). Mice were euthanised 4 h after injection of the ^99m^Tc-p5+14. The image data for the low and high energy radionuclides were obtained sequentially by acquiring 60, 16-second projections in a total of 1.5 revolutions. A 1 mm-diameter aperture, five-pinhole (mouse whole body) collimator was used at 30 mm from the centre of the field of view. The image data in the appropriate energy window were reconstructed using a Maximum A Priori algorithm (MAP - 16 iterations, 6 subsets, β = 1) onto a matrix with x and y dimensions of 88 and z dimension of 312 and 0.5-mm isotropic voxels. Post hoc attenuation correction was then applied to reconstructed data using CT image data. Scatter correction was performed using a SPECT triple energy window (TEW) method^38^.

For all mice, CT data were acquired for anatomic co-registration and attenuation correction, using an x-ray voltage biased to 80 kVp with a 500 μA anode current. Two bed positions were used with 240 ms exposure and 361 projections collected covering 360° of continuous rotation with low magnification and binning at 4. The data were reconstructed using an implementation of the Feldkamp-filtered cone beam algorithm^39^ onto a 256 × 256 × 603 matrix with isotropic 211.4-μm voxels.

### Histological evaluations

For histological evaluation of the presence of amyloid in each mouse, 6- μm-thick sections were cut from formalin-fixed, paraffin-embedded blocks containing tissues from mice that had received radiolabelled SAP and p5+14. The tissue sections were placed on Plus microscope slides and counterstained with hematoxylin and eosin (H&E). Detection of amyloid was achieved in consecutive tissue sections by staining with an alkaline Congo red solution (0.8% w/v Congo red, 0.2% w/v KOH, 80% ethanol) for 1 h at room temperature followed by counterstain with Mayer’s hematoxylin for 2 min.

All samples were examined using a Leica DM500 light microscope fitted with cross-polarising filters (for Congo red). Digital microscopic images were acquired using a cooled CCD camera (SPOT RT-Slider; Diagnostic Instruments).

### Dual-energy biodistribution measurements

For comparison of the p5+14 and p5 peptides, mice were injected IV in the lateral tail vein with a mixture of ^125^I-p5+14 (150 μCi, ~3 μg) and ^99m^Tc-p5 (300 μCi, ~8 μg) and the mice euthanised by an overdose of isoflurane anaesthetic 1 h post injection. Tissues were harvested at necropsy for biodistribution measurements. Organs were also harvested from mice injected with ^125^I-SAP and ^99m^Tc-p5+14 after SPECT/CT imaging. A small volume of tissue from each mouse was placed into a tared plastic vial and weighed. The radioactivity in the low and high energy windows (^125^I and ^99m^Tc, respectively) was measured using an automated Wizard 3 gamma counter (1480 Wallac Gamma Counter, Perkin Elmer) with a crossover correction from the high to low energy channel of 4.6%, applied manually. Biodistribution data were expressed as percent injected dose per gram (%ID/g) of tissue.

### *In vitro* peptide-amyloid binding assay

Binding of ^125^I-labelled p5 and p5+14 to synthetic amyloid fibrils composed of recombinant λ6 variable domain proteins derived from patient Wil (rVλ6Wil) and human islet amyloid polypeptide as well as human light chain associated (AL) amyloid extracts and murine AA liver homogenates, were performed as previously described^15^,^18^. Briefly, 25 μL of 1 mg/mL substrate was centrifuged in a 0.5 mL microfuge tube at 21,000 × g for 5 min. The supernatant was discarded and pellet resuspended in 200 μL of PBS with 0.05% tween-20 (PBST). Ten microliters of a 1:100 dilution of ^125^I-labelled peptide (~100,000 counts per minute (CPM); ~5 ng peptide) was added to the suspension. The mixture was rotated at RT for 1 h. Samples were then centrifuged twice at 15,000 × g for 10 min. Supernatants and pellets were separated after each step, and the radioactivity in each was measured using a Cobra II gamma counter (Perkin Elmer) with a 1 min acquisition. Binding assays were performed in PBS or solutions of increasing ionic strength (0.15–2 M NaCl), and the percentage of ^125^I-labelled peptide bound to pelleted substrate was determined using “equation (1)”:


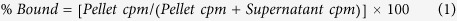


### Statistical Methods

Skewness and kurtosis statistics were used to assess the statistical assumption of normality for between-subjects comparisons and correlations. Any skewness or kurtosis statistic above an absolute value of 2.0 assumed a non-normal distribution. The assumption of homogeneity of variance was tested with Levene’s Test of Equality of Variances. Independent samples t-tests were used to compare p5+14 and SAP retention in amyloid laden organs (liver, spleen, pancreas, intestines and heart). Means and standard deviations were reported for the analyses. If a statistical assumption was violated, a non-parametric Mann-Whitney U test was used. Medians and interquartile ranges (IQR) were reported for non-parametric statistics. Pearson correlations were run to compare tissue biodistribution data (%ID/g) in all organs. To adjust for multiple comparisons, a Bonferroni corrected alpha value of 0.01 (alpha value of 0.05/5 tests = 0.01) was used to determine statistical significance and all analyses were conducted using SPSS Version 21 (IBM Corp., Armonk, NY).

## Additional Information

**How to cite this article**: Martin, E. B. *et al*. Comparative evaluation of p5+14 with SAP and peptide p5 by dual-energy SPECT imaging of mice with AA amyloidosis. *Sci. Rep.* **6**, 22695; doi: 10.1038/srep22695 (2016).

## Figures and Tables

**Table 1:** The amyloid reactivity, *in vitro*, of peptides p5 and p5+14.

**Substrate**	^**125**^**I-p5(% bound ± SD)**	^**125**^**I-p5+14(% bound ± SD)**	**Significance**^1^
AA liver homogenate	70.2 ± 0.64	80.3 ± 0.21	p = 0.002
rVλ6Wil (AL) fibrils	74.6 ± 3.04	89.4 ± 0.00	p = 0.02
ALκ4 amyloid extract	56.1 ± 0.78	79.6 ± 0.21	p = 0.0006
IAPP fibrils	21.2 ± 0.99	42.9 ± 0.07	p = 0.001

^1^Statistical analysis was performed using an unpaired *t*-test.

**Table 2:** Statistical analysis of ^125^I-SAP and ^99m^Tc-p5+14 in AA mice (*n* = 5).

**Organ**	**Correlationcoefficient**^1^	**Correlationsignificance**	^**99m**^**Tc-p5+14Mean ± SD**	^**125**^**I-SAPMean ± SD**	**Significance**(***p***** < 0.01)**^2^
Liver	0.97	*p* = 0.005	7.4 ± 1.9	14.1 ± 3.5	*p* = 0.006
Spleen	0.77	*NS*	5.0 ± 1.0	23.8 ± 3.5	*p* < 0.001
Pancreas	0.92	*p* = 0.030	7.0 (3.2)^3^	0.7 (0.5)^3^	*p* = 0.009^4^
Intestine	0.87	*NS*	4.2 ± 2.3	0.8 ± 1.1	*NS*
Heart	0.05	*NS*	1.8 ± 0.2	0.7 ± 0.1	*p* < 0.001

^1^Pearson correlation analysis.

^2^Independent sample t-test.

^3^Median (Interquartile range).

^4^Non-parametric Mann Whitney U test. *NS*, not significant.
